# Advancements in prosthetics and orthotics: overview of the *Journal of NeuroEngineering & Rehabilitation’s* special topic edition

**DOI:** 10.1186/s12984-018-0399-2

**Published:** 2018-09-05

**Authors:** M. Jason Highsmith, Phil M. Stevens, Michael S. Orendurff, Andreas Kannenberg

**Affiliations:** 1 Extremity Trauma & Amputation Center of Excellence (EACE), U.S. Department of Veterans Affairs, Tampa, FL USA; 2 School of Physical Therapy and Rehabilitation Sciences, University of South Florida, Tampa, FL USA; 3 Department of Clinical and Scientific Affairs, Hanger Clinic, Austin, TX USA; 4 Department of Physical Medicine and Rehabilitation, University of Utah, Salt Lake City, UT USA; 5 Motion & Sports Performance Laboratory, Stanford Childrens Health, Palo Alto, CA USA; 6 Department of Clinical Research & Services, Ottobock Healthcare, Austin, TX USA

The Journal of Neuroengineering and Rehabilitation (JNER. https://jneuroengrehab.biomedcentral.com/), a BioMed Central journal partner, is pleased to present the enclosed scientific findings in prosthetics and orthotics. The included papers were identified by the scientific content committee from the American Orthotic and Prosthetic Association’s (AOPA) clinical content committee for the 2017 AOPA World Congress (September 2017. Las Vegas, NV. USA), as the top scientific presentations.

Presenters of the identified papers were invited to submit their full manuscript to the JNER for rigorous peer-review and consideration for publication in a special topic edition on prosthetics and orthotics that would capture the essence of the 2017 World Congress (http://www.aopanet.org/education/aopa-world-congress/). The goal of this special topics edition was to afford the scientific and clinical communities the opportunity to take a “deeper dive” into the detail of the top presentations of the 2017 AOPA World Congress. These topics were regarded as cutting-edge topics ranging from exercise testing and cardiovascular events in patients with limb loss to gait assessment and novel therapies such as use of a virtual environment during rehabilitation. Additionally and importantly, economic evaluations for orthotic and prosthetic devices are also included.

Numerous stakeholders are responsible for facilitating development of this special issue of JNER. The guest editorial board wishes to thank AOPA and its volunteer content committee for vetting and identifying its top papers and presenters, the American Board for Certification in Orthotics, Prosthetics and Pedorthics, Inc. for its generous sponsorship, to the authors for submitting their work and of course to the JNER team for their partnership. It is our hope that the entire community of stakeholders in prosthetics and orthotics benefit from the findings contained in this edition of the JNER.

We hope the findings will contribute meaningfully to the body of knowledge within prosthetics and orthotics and that they will assist in the reimbursement arena with clinical decision making and that others will build on and expand the findings and further push the boundaries of what can be achieved for those who use prosthetics and orthotics.

